# Cynaroside inhibits *Leishmania donovani* UDP-galactopyranose mutase and induces reactive oxygen species to exert antileishmanial response

**DOI:** 10.1042/BSR20203857

**Published:** 2021-01-12

**Authors:** Shams Tabrez, Fazlur Rahman, Rahat Ali, Abdulaziz S. Alouffi, Sajjadul Kadir Akand, Bader Mohammed Alshehri, Fahdah Ayed Alshammari, Aftab Alam, Mohammed A. Alaidarous, Saeed Banawas, Abdul Aziz Bin Dukhyil, Abdur Rub

**Affiliations:** 1 Infection and Immunity Lab (414), Department of Biotechnology, Jamia Millia Islamia (A Central University), New Delhi 110025, India; 2 King Abdulaziz City for Science and Technology, Riyadh, 11442, Saudi Arabia; 3 Department of Medical Laboratory Sciences, College of Applied Medical Sciences, Majmaah University, Al Majmaah 11952, Saudi Arabia; 4 College of Sciences and Literature Microbiology, Nothern Border University, KSA; 5 Centre for Interdisciplinary Research in Basic Sciences, Jamia Millia Islamia (A Central University), New Delhi 110025, India; 6 Health and Basic Sciences Research Center, Majmaah University, Al Majmaah, Saudi Arabia; 7 Department of Biomedical Sciences, Oregon State University, Corvallis, OR 97331, U.S.A.

**Keywords:** amastigote, Cynaroside, IC50, Leishmaniasis, parasite, promastigote

## Abstract

Cynaroside, a flavonoid, has been shown to have antibacterial, antifungal and anticancer activities. Here, we evaluated its antileishmanial properties and its mechanism of action through different *in silico* and *in vitro* assays. Cynaroside exhibited antileishmanial activity in time- and dose-dependent manner with 50% of inhibitory concentration (IC_50_) value of 49.49 ± 3.515 µM *in vitro*. It inhibited the growth of parasite significantly at only 20 µM concentration when used in combination with miltefosine, a standard drug which has very high toxicity. It also inhibited the intra-macrophagic parasite significantly at low doses when used in combination with miltefosine. It showed less toxicity than the existing antileishmanial drug, miltefosine at similar doses. Propidium iodide staining showed that cynaroside inhibited the parasites in G_0_/G_1_ phase of cell cycle. 2,7-dichloro dihydro fluorescein diacetate (H_2_DCFDA) staining showed cynaroside induced antileishmanial activity through reactive oxygen species (ROS) generation in parasites. Molecular-docking studies with key drug targets of *Leishmania donovani* showed significant inhibition. Out of these targets, cynaroside showed strongest affinity with uridine diphosphate (UDP)-galactopyranose mutase with −10.4 kcal/mol which was further validated by molecular dynamics (MD) simulation. The bioactivity, ADMET (absorption, distribution, metabolism, excretion and toxicity) properties, Organisation for Economic Co-operation and Development (OECD) chemical classification and toxicity risk prediction showed cynaroside as an enzyme inhibitor having sufficient solubility and non-toxic properties. In conclusion, cynaroside may be used alone or in combination with existing drug, miltefosine to control leishmaniasis with less cytotoxicity.

## Introduction

Leishmaniasis is a vector-borne disease caused by obligate intra-cellular protozoan parasite of genus *Leishmania*. The disease is endemic in more than 89 different countries worldwide. There are 20 different species of *Leishmania* which can infect animals, humans and sand-flies to cause species-specific complications in the host. The majority of *Leishmania* species responsible for causing leishmaniasis are *Leishmania major, L. infantum, L. chagasi* and *L. donovani*. The reservoirs of the disease are animals like canines and rodents (zoonotic cycle) and human (anthroponotic cycle) [1,2]. There are several molecules which are reported to have anti-leishmanial activity yet, but only a few are classified as effective antileishmanial drugs that are used for the treatment of leishmaniasis [3]. The first effective drug used for this purpose was ureastibamine. The refinement and development of pentavalent antimonials reduced the side effects and are used for treating all forms of leishmaniasis these days. Later on, pentamidine isethionate and amphotericin B were started in use [4]. Due to the increasing incidence of drug resistance, toxicity, less availability and high cost, there is an urgent need to search for alternative therapies against drug targets of *Leishmania*. Flavonoids consist of a large group of polyphenolic compounds having a benzo-γ-pyrone structure and are ubiquitously present in plants. They are synthesized by the phenylpropanoid pathway. Available reports tend to show that secondary metabolites of phenolic nature including flavonoids are responsible for the variety of pharmacological activities [5–7]. There are several reports about the protective role of flavonoids against human diseases including protozoal infections [5,8,9]. Cynaroside is also known as 7-glucoluteolin which belongs to flavonoid. It is present in dandelion coffee-*Ferulavaria, F. foetida* and in *Campanula persicifolia, C. rotundi folia* and *C. acutofolia* [10]. Cynaroside is also present in perennial plant *Anthriscus sylvestris* and demonstrates biological activity especially against Gram-negative bacteria, shows anti-mutagenic activity, inhibits biofilm formation of *Pseudomonas aeruginosa* and *Staphylococcus aureus* [11]. Cynaroside has been shown to reduce the cisplatin-induced toxicity of kidney and oocytes *in vitro* as well as *in vivo* [12]. It reduced the nephrotoxicity and ootoxicity through the suppression of apoptosis, restoration of mitochondrial dysfunction and caspase-3 activation [12]. Cynaroside has been reported as one of the main chemical constituents of *Cynara scolymus* also known as artichoke [13]. It is extensively cultivated in Mediterranean region, African and American countries. *C. scolymus* sprout is commonly used as a vegetable and its leaves are normally used for the treatment of dyspeptic, hepatitis, hyperlipidemia and obesity disorders [14]. It has anti-inflammatory and antioxidant activities. It also induces apoptosis in different types of cancer cell lines [15,16].

There are several enzymes in the life cycle of *L. donovani* which are very important for its growth, survival, proliferation and pathogenesis inside the host. Few of these are folate and polyamine biosynthesis pathway enzymes. Pteridine reductase1 (PTR1) is an NADPH-dependent short-chain reductase responsible for the salvage of pterins in the protozoan parasite *Leishmania*. This enzyme acts as a metabolic bypass for drugs targeting dihydrofolate reductase [17]. UDP-galactopyranose mutase (UGM) is a flavo-dependent enzyme. It catalyzes the conversion of uridine diphosphate (UDP)-galactopyranose into UDP-galactofuranose. UGM is the only source for the biosynthesis of a sugar galactofuranose (Galf) and it is not present in a mammalian host which makes it an ideal drug target against *L. donovani* [18]. Pyruvate phosphate dikinase (PPDK) is one of the key players for the entry of alanine in intracellular amastigotes. PPDK performs the reversible conversion of PPi, AMP and phosphoenolpyruvate (PEP) into Pi, ATP and pyruvate, respectively. The absence of PPDK in mammals and its essential role in parasites makes this enzyme an attractive target for designing antileishmanial drug [19]. The major thiol of *L. donovani* is synthesized by trypanothione synthetase which maintains redox potential essential for the survival of the parasite. Trypanothione is synthesized by trypanothione synthetase enzyme in *L. donovani*. It is absent from humans. Therefore, this enzyme can also be targeted for the development of drug against leishmaniasis [20]. Keeping the need of the time in consideration, we planned to study the role of antileishmanial effect of cynaroside through *in vitro* assays and also tried to workout its molecular mechanism of action through different *in vitro* as well as *in silico* experiments.

## Materials and methods

### Chemicals and reagents

M199 media for promastigote culturing, Roswell Park Memorial Institute (RPMI) 1640 media for cell line, penicillin–streptomycin antibiotic cocktail, fetal bovine serum (FBS) were purchased from Gibco, Thermo Fisher Scientific. HEPES, sodium bicarbonate and paraformaldehyde were procured from Sigma–Aldrich. Miltefosine, 3-(4,5 dimethyl- thiazol-2-yl)-2,5-diphenyl tetrazolium bromide (MTT) assay reagents and cell culture-grade dimethyl sulfoxide (DMSO) were purchased from Merck & Co., Inc. Propidium iodide, RNase A and reactive oxygen species (ROS) dye (H_2_DCFDA) were procured from Thermo Scientific. Cynaroside was purchased from ChemScene India. All the other chemicals and reagents were purchased from Sigma–Aldrich or Merck, unless stated otherwise.

### Parasite and THP-1 cell culture

Infective strain of *L. donovani* (MHOM/IN/83/AG83) was maintained in M199 media at pH 7.4 and supplemented with 25 mM HEPES, 10% heat-inactivated FBS and 1% penicillin–streptomycin antibiotic cocktail and maintained at 22°C. THP-1 human monocytic cell line was maintained in RPMI 1640 media supplemented with 10% FBS and 1% penicillin–streptomycin antibiotic in 5% CO_2_ at 37°C. THP-1 monocytic cells were stimulated with 20 ng/ml of phorbol myristate acetate (PMA) for differentiation into macrophages.

### Anti-promastigote evaluation and IC_50_ determination

Log-phase promastigotes were enumerated and incubated at the density of 5 × 10^6^ parasites in the absence and presence of cynaroside at two-fold serial dilutions starting at 120 µM for 48 h at 22°C. The percentage parasite viability was calculated as (Mean parasite number of treated sample/Mean parasite of control) × 100. The 50% inhibitory concentration was determined by extrapolating the graph of % viability of parasites against concentration of the drug/compound. Morphological changes of the parasites were observed under 20× and 40× lenses using LMI U.K. vImage software.

### Anti-amastigote and cytotoxicity evaluation of cynaroside

A total of 2 × 10^6^ THP-1 differentiated macrophages were plated in 96-well tissue culture-grade plates in RPMI 1640 complete media with 5% CO_2_ at 37°C to evaluate the cytotoxic activity of cynaroside. Differentiated adherent cells were washed with plain RPMI 1640 media and exposed to two-fold serial dilution of cynaroside starting from 500 µM for 24 h. Cells were further incubated with 50 µM of 5 mg/ml of MTT for 3–4 h, thereafter the resulting formazan was dissolved in 150 µM of DMSO. The amount of formazan produced represented the relative number of viable cells which was recorded spectrophotometrically at 570 nm by ELISA plate reader. The cytotoxic concentration 50%, i.e. CC_50_ value was determined by extrapolation of the dose–response curve of percentage cell viability vs concentration of the compound. For parasite load calculation, THP-1-differentiated macrophages were plated on coverslip in six-well plates and infected by *L. donovani* with 1:10. Infected macrophages were treated with different concentrations of the drugs/compounds for 48 h and then cells were fixed by chilled methanol and stained by Giemsa to calculate the % parasites load.

### Study of cell cycle of promastigotes

In brief, the promastigotes were cultured in the absence and presence of different concentrations of cynaroside and miltefosine as the positive control. The promastigotes were harvested after 48 h of incubation and washed thrice with PBS followed by fixation with 80% chilled ethanol and kept at 4°C overnight. The fixed cells were washed twice with PBS and incubated with 200 μg/ml of RNase at 37°C for 1 h followed by staining with 50 µM of 1 mg/ml of propidium iodide (PI) for 20 min in dark. Cells were analyzed through flow cytometer (BD FACS ARIA).

### ROS estimation

To assess the cynaroside-induced ROS generation, 5 × 10^6^ parasites were incubated with different concentrations of compounds/drugs at 22°C for 48 h. The treated parasites were washed with PBS and incubated with 10 µM of fluorescent dye, 2,7-dichloro dihydro fluorescein diacetate (H_2_DCFDA) for 20 min in dark and analyzed through BD FACS ARIA. Data were represented in the form of histograms.

### Homology modeling

The crystal structures of Pteridine reductase protein was retrieved from Protein Data Bank (PDB) [ID: 2XOX (PTR1)]. The PDB file used for docking-based virtual screening study was prepared by removing water molecules and adding hydrogen atoms. Due to the lack of solved 3D structure of *Ld*UGM, *Ld*PPDK and *Ld*TS, homology modeling was opted to determine the structure of these enzymes. The protein sequence of these enzymes was retrieved from the NCBI protein database and PSI-BLAST was performed against PDB to find identical protein [21]. Homology modeling for the proteins was performed by SWISS-MODEL homology modeling server [22]. The template structures generated were assessed and the suitable template was selected for modeling the 3D structure. Out of all the modeled structures, one of the modeled structures was selected based on the sequence identity, Q-Mean, GMQE values and Ramachandran plot, and validated and analyzed through the RAMPAGE, PROCHECK and PDBsum servers [21,23]. The sequence identity between the target and template structure and the root-mean-square deviation (RMSD) value was calculated in Chimera [24].

### Protein processing and ligand preparation

The downloaded and modeled protein structures were prepared by ‘Discovery Studio visualizer 2020’. Protein molecules were loaded and the water molecules present in the proteins were removed and polar hydrogen atoms were added. The ligands attached to the proteins were selected and binding sites were defined followed by removing the ligands. The identification of the important residues of the catalytic pockets were taken from the native binding pockets of accessible crystal structure of proteins, various submitted literatures, from their homologous template proteins and investigation within the mechanism of inhibition. The active site residues of the protein are found and noted down. Preparation of ligand molecule was performed using PyRx.

### Molecular docking and molecular dynamics simulation of protein–ligand complex

The protein molecule was loaded in the PyRx Virtual Screening Tool [25] and was converted into a macromolecule pdbqt format. The cynaroside to be screened was imported and converted into the ligand pdbqt format. In the Vina wizard the pdbqt macromolecules and pdbqt ligands are selected and the grid box is set up with all the active site residues containing within the grid box. The docking is performed by running Vina. The output file was analyzed to find binding energy. The best orientation of the cynaroside was selected and saved as a PDB file. The protein molecule and the best-oriented ligand molecule were loaded in Pymol [19] and the protein–ligand complex was visualized. The protein–ligand complex was loaded in Ligplot software [26] and the output 2D diagram was analyzed to find the number of hydrogen bonds and the binding site residues of the protein.

The most favourable binding poses of the cynaroside were analyzed by choosing the lowest free energy of binding (ΔG) and the lowest inhibition constant (*K*_i_) which is calculated using the following formula:

$$\text{Ki}=\frac{\exp(\Delta \text{G}*1000)}{\text{RT}}$$

where ΔG = docking energy; R = 1.98719 cal.K^−1^.mol^−1^; T = 298.15 K, *K*_i_ = inhibition constant (nM).

Additionally, we have performed molecular dynamics (MD) simulations using the GROMACS (version 5.1) software [18,24,25] for the best docking complex at 50 ns at 300 K. The resulting trajectories were analyzed, using RMSD, RMSF, RG and SASA by the utilities provided by GROMACS.

### Pharmacokinetics studies

The selected ligand was evaluated for pharmacological profiles by analyzing for Lipinski’s rule of violation-5, which was analyzed by Molsoft L.L.C.: Drug-Likeness and molecular property prediction for drug-likeness (http://www.molsoft.com/mprop/) [27]. The bioactivity of cynaroside was checked by Molinspiration (https://molinspiration.com/cgi-bin/properties). The cynaroside was further evaluated for ADMET (absorption, distribution, metabolism, excretion and toxicity) properties by GUSAR [28] and SwissADME database [29]. SwissADME software helps us to identify the selected drug molecule by applying different virtual screening methods. Different components of lipophilicity (iLOGP, WLOGP, XLOGP3, MLOGP, Log Po/w), pharmacokinetics (GI absorption, BBB permeant, P-gp substrate, Log (*K*_p_)), water solubility also helped in the preliminary testing of the suitable drug molecule. OSIRIS Property Explorer programme was used to evaluate the mutagenic, tumorigenic, irritant and reproductive risks, and which also provides information on the compound’s toxicity, solubility (LogS), hydrophilicity (LogP), molecular weight, drug-likeness and drug score [30].

### Statistical analysis

All the experiments were performed in technical triplicate and done at least thrice, and the results represented are the mean of the triplicate with SEM. Statistical analysis was performed using GraphPad Prism 7.0 software and the statistical significance was calculated using one-way ANOVA followed by Dunnett’s multiple comparison test. *P*<0.5 was considered statistically significant.

## Results and discussion

### Cynaroside inhibits growth and survival of promastigotes *in vitro*

**Figure 1:**
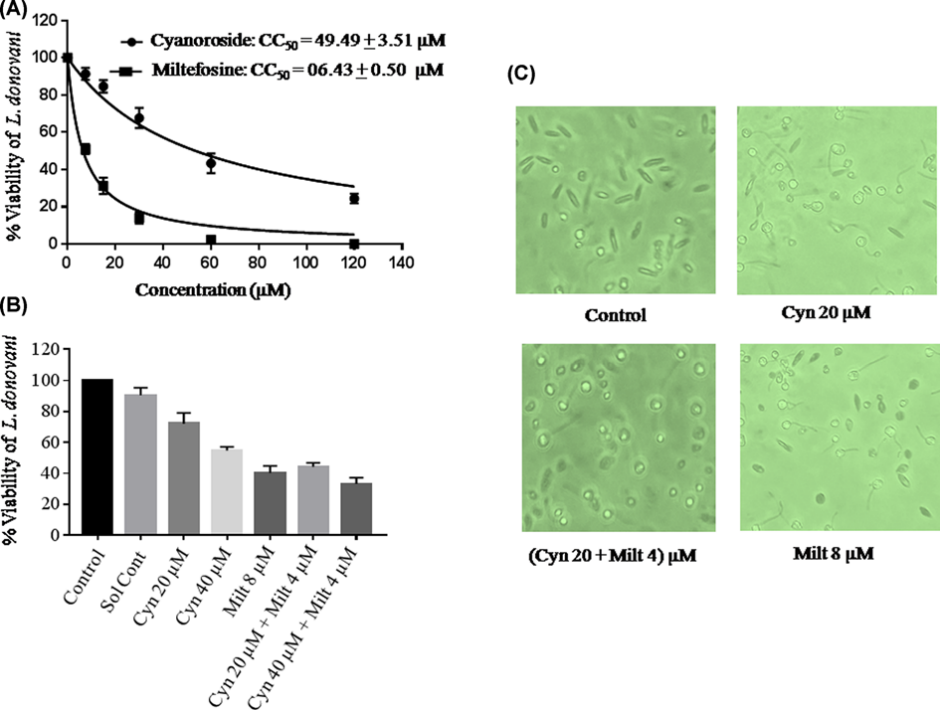
Antileishmanial effects of cynaroside and IC_50_ determination (**A**) *L. donovani* promastigotes were treated with different concentration of cynaroside, with miltefosine as standard drug and control (without any treatment). IC_50_ was determined as described in the methods. (**B**) Parasites were grown in cynaroside, miltefosine and both in combination and percentage inhibition is plotted vs concentration. (**C**) Morphological changes in parasite on treatment with different compounds as mentioned in figure.

The cynaroside treatment showed the dose-dependent inhibition of parasite growth with 50% of inhibitory concentration (IC_50_) of 49.49 ± 3.515 µM (Figure 1A). Though, antileishmanial drug miltefosine showed similar trend with IC_50_ value of 6.439 ± 0.5032 µM (Figure 1A). Cynaroside inhibited only 20% of parasites at 20 µM concentration though it inhibited more than 50% of parasites when used in combination with non-toxic dose of miltefosine, 4 µM (Figure 1B). The morphological analysis of treated *L. donovani* shows a characteristic difference as compared with untreated parasites. Parasites were visualized as cylindrical, elongated and flagellated in untreated control though oval and reduced size with degenerated flagella in treated samples (Figure 1C).

### Cynaroside inhibited intra-macrophagic parasites with less cytotoxicity

**Figure 2:**
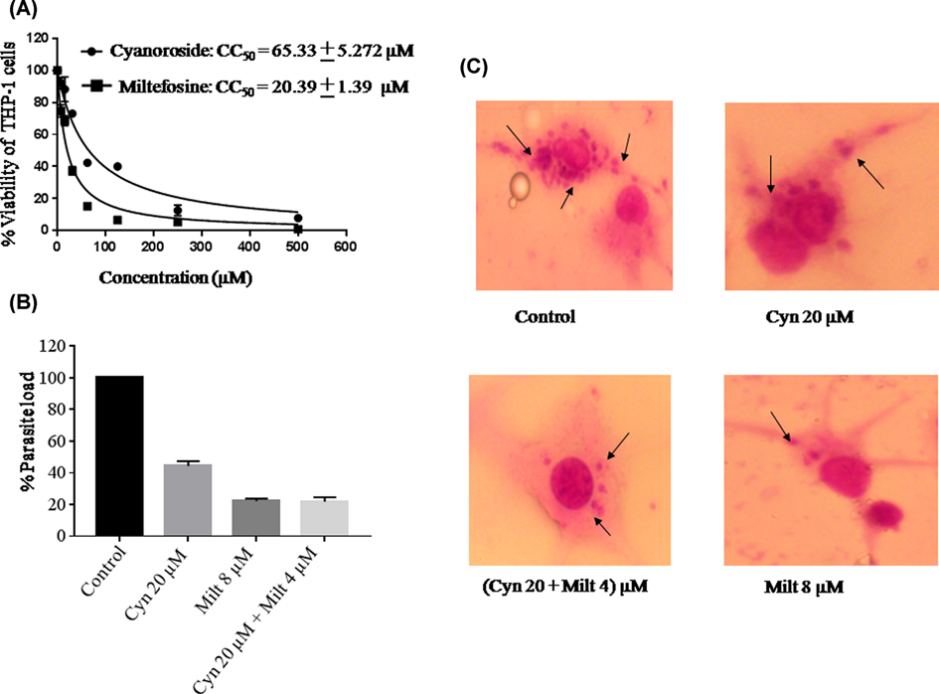
Cynaroside reduced the intra-macrophagic amastigotes (**A**) THP-1-differentiated macrophages were treated with different concentrations of cynaroside and miltefosine (0–500 μM) and cell viability was assessed by MTT assay. (**B**) THP-1-differentiated macrophages were parasitized in 1:10 ratio with stationary phase promastigotes and then treated with cynaroside, miltefosine and cynaroside in combination with miltefosine. Percent reduction in the parasite load was determined as described in the ‘Materials and methods’ section. *P*<0.05, value was statistically significant as compared with control. (**C**) *L. donovani* infected macrophages were stained with Giemsa and parasites were observed inside the macrophages. The images were captured at 100× under oil immersion. The arrow indicates internalized parasites.

Cell cytotoxicity of cynaroside along with the miltefosine as the positive control was evaluated against THP-1-differentiated macrophages. The macrophages were incubated at different concentrations of cynaroside, miltefosine and both in combination. The cell viability was assessed through MTT assay and 50% cell cytotoxic (CC_50_) concentration of cynaroside was determined as 65.33 ± 5.272 µM while that of miltefosine as 20.39 ± 1.69 µM (Figure 2A). Infected macrophages were treated with cynaroside, miltefosine and both in combination at the least cytotoxic dose to assess the effect of cynaroside on the intra-macrophagic amastigotes (Figure 2B). Cynaroside reduced the number of intra-macrophagic amastigotes by 50% at 20 µM concentration though it reduced to 80% when used in combination with 4 µM miltefosine (Figure 2B). Similar effects were visualized in the images of stained slides (Figure 2C). We have used the higher concentration of cynaroside because it is less toxic up to higher doses in comparison with miltefosine.

### Cynaroside checks the parasites in G_0_/G_1_ phase of cell cycle

**Figure 3:**
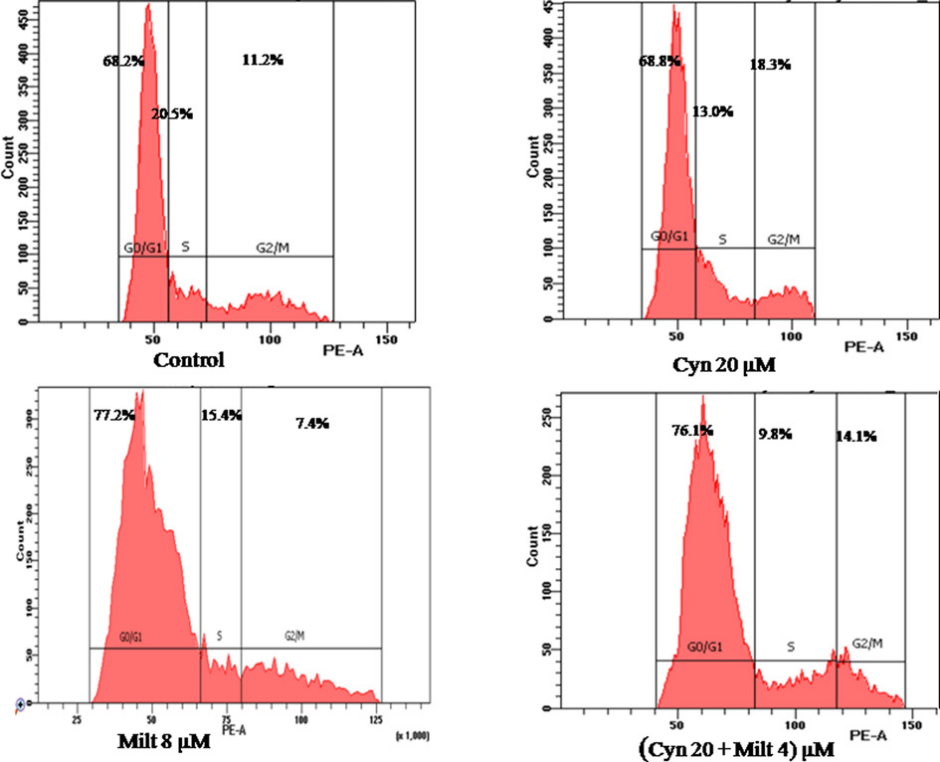
Cynaroside checks the parasites in G_0_/G_1_ phase of cell cycle *L. donovani* promastigotes were treated with cynaroside, miltefosine and both in combination for 48 h at 22°C. The treated samples were processed for cell cycle and acquired through BD FACS ARIA flow cytometer.

Cell cycle analysis portrays the effect of treatments on its progression in different stages. Cynaroside arrested the promastigotes progressing from S to G_2_/M at 20 µM concentration (Figure 3). There was approximately 7% surge in S phase population when both cynaroside (20 µM) and miltefosine (4 µM) were used in combination (Figure 3). It suggested that cynaroside in combination with low dose of miltefosine inhibited the cell cycle progression as does higher dose of miltefosine. Higher doses of miltefosine are highly cytotoxic and can not be used safely.

### Cynaroside treatment induced ROS generation

**Figure 4:**
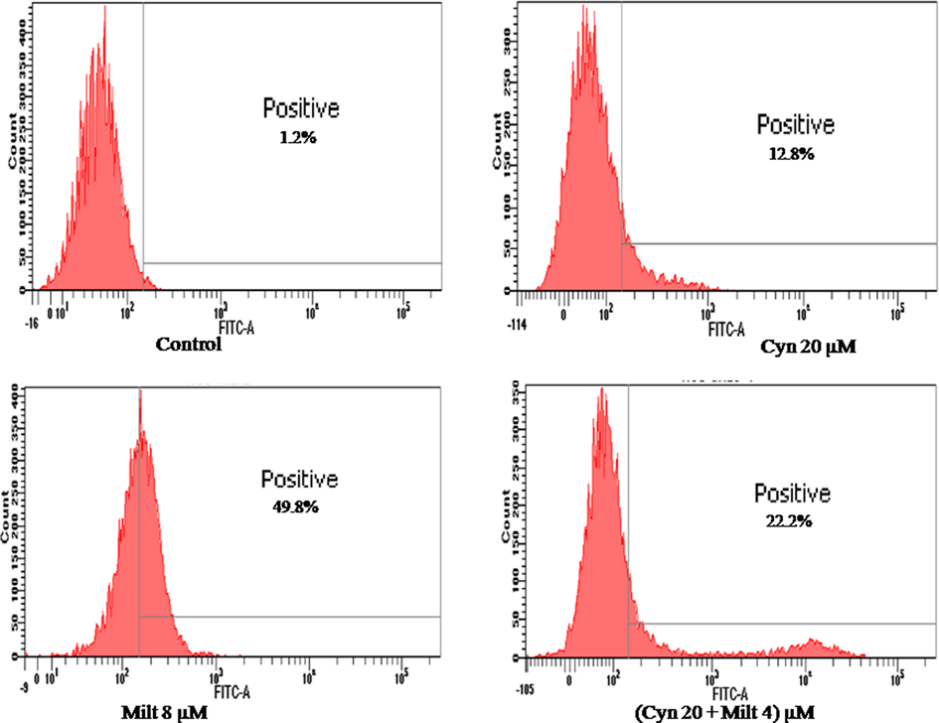
Cynaroside induced ROS generation in parasites *L. donovani* promastigotes were incubated with cynaroside for 48 h and stained with 10 µM H_2_DCFDA, then samples were acquired through BD FACS ARIA flow cytometer.

To study the impact of cynaroside treatment in inducing ROS generation in *L. donovani* promastigotes, H_2_DCFDA was used which is fluorescent green in presence of OH radicals and H_2_O_2_. Fluorescence intensity is directly proportional to ROS generation and peak shifting to right. In untreated control no green fluorescence was observed though at 20 µM of cynaroside the peak was shifted to right and approximately 12.8% of cells showed ROS production (Figure 4). On the other hand cynaroside in combination with low dose of miltefosine induced the ROS generation in approximately 22.2% of cells (Figure 4). The result suggested, cynaroside treatment induced ROS production that lead to stress in the parasites. Cynaroside in combination with low dose of miltefosine was found to be more effective than alone (Figure 4).

### Cynaroside blocks important drug targets of *L. donovani*

Molecular docking is one among the foremost popular methods within the field of computer-aided drug designing (CADD) for the identification of latest drug leads [31,32]. In the present time, CADD is getting used to annotate and analyze big drug libraries rapidly and hence saving an enormous amount of energy, time and costs [33]. Among all kinds of various interactions like amide–π interactions, π–π, H-bond etc., the binding efficiency is being evaluated on the idea of hydrogen bonding [31,34]. First, the 3D structures of the enzymes were constructed and validated by homology modeling from template structures (Supplementary Figures S1 and S2 and Tables S1–S3). Molecular docking is used to find cynaroside as a potentially active inhibitor against *Ld*UGM, *Ld*PTR1, *Ld*PPDK and *Ld*TS enzymes of *L. donovani* (Supplementary Figure S4). On the basis of binding affinity, cynaroside has been found to possess binding energy of −10.4 , −8.1, −7.8 and−7.7 kcal/mol, with of *Ld*UGM, *Ld*PTR1, *Ld*PPDK and *Ld*TS enzymes, respectively (Table 1). The binding energy (kcal/mol) is employed to compare and study the binding affinity of various compounds/ligands with their respective target molecule, i.e. lower the binding energy, higher the affinity of the ligand for the receptor. So, the ligand with the highest affinity can be chosen as the potential drug for further studies.

**Table 1:** Binding of cynaroside with key proteins of *L. donovani*

Sl. No.	Enzymes	Binding affinity (kcal/mol)	Number of Hydrogen bonds	p*K*ipred (μM)	Binding site residues
1.	UGM	−10.4	9	7.65	Val^61^, Tyr^98^, Tyr^157^, Arg^177^, Tyr^328^, Arg^338^, Tyr^407^, Tyr^442^(2)
2.	Pteridine reductase	−8.1	4	5.96	Ser^40^(3), Ser^146^
3.	PPDK	−7.8	8	5.74	Asn^366^, Gly^367^, Arg^369^(3), Arg^395^, Gln^400^, Lys^897^
4.	Trypanothione synthetase	−7.7	3	5.66	Glu^44^(2), Gln^322^

**Figure 5:**
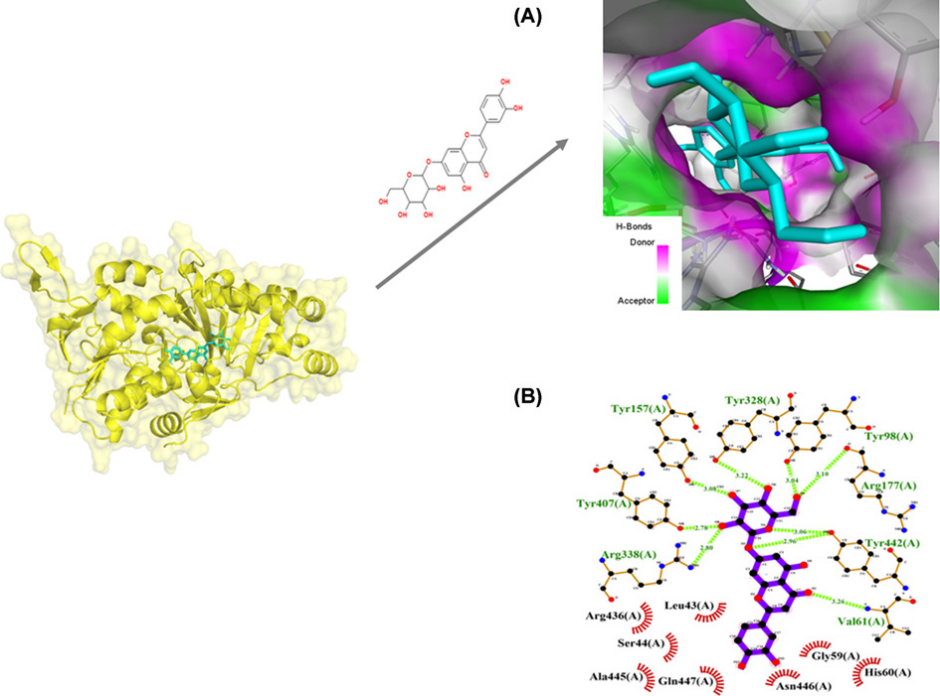
*In silico* binding study of cynaroside with *Ld*UGM (**A**) Molecular view of cynaroside (stick figure in cyan color) in the binding pocket. (**B**) 2D view of cynaroside docked in with UDP galactopyranose as LigPlot figure. Amino acid residues forming hydrophobic interactions were highlighted in red circles. Amino acids contributing to hydrogen bonds were labeled dark green and hydrogen bonds were indicated as dotted lines with bond length labeled in lime green.

**Figure 6:**
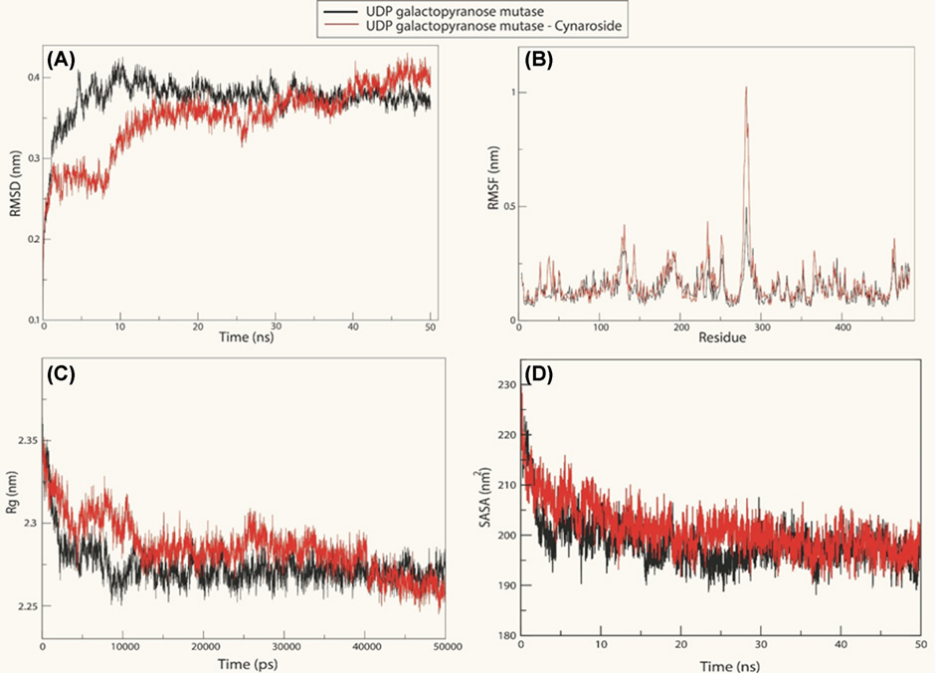
MD simulation of *Ld*UGM with cynaroside Structural analysis of the ***Ld*UGM** (Black) and cynaroside complexes (Red) were taken from the trajectory files with the built-in function of GROMACS 5.1.1. (**A**) RMSD of backbone atoms. (**B**) RMSF of the of ***Ld*UGM** and cynaroside complexes versus time at 300 K. (**C**) Radius of gyration of ***Ld*UGM** and cynaroside complexes versus time at 300 K. (**D**) Solvent-accessible surface area (SASA) of ***Ld*UGM** and cynaroside complexes versus time at 300 K.

The enzyme UGM shows the lowest binding energy. The binding pattern of cynaroside with *Ld*UGM may hamper the substrate accessibility and its subsequent inhibition as shown in (Figure 5A) where the binding energy is −10.4 kcal/mol (Table 1). Cynaroside interacts with Val^61^, Tyr^98^, Tyr^157^, Arg^177^, Tyr^328^, Arg^338^, Tyr^407^, Tyr^442^(2) binding site residues of *Ld*UGM by forming nine intermolecular hydrogen bonds with bond length ranging from 3.26, 3.10, 3.08, 3.10, 3.22, 2.80, 2.78, 2.96 and 3.26 Å, respectively, as shown in Ligplot (Figure 5B). It has been observed that residues of *Ld*UGM such as Leu^43^, Ser^44^, Gly^59^, His^60^, Arg^436^, Ala^445^, Asn^446^, Gln^447^ (*n*=8) are showing significant interactions with cynaroside (Figure 5B). The enzyme *Ld*PTR1 shows binding to cynaroside by forming four hydrogen bonds with residues Ser^40^(3) and Ser^146^ and other significant hydrophobic interactions formed via Gly^13^, Ala^15^, His^36^, Tyr^37^, His^38^, Arg^39^, Asp^65^, Ser^67^, Ala^110^, Ser^111^ and Leu^143^ (*n*=11) as shown in Ligplot (Supplementary Figure S5A). Cynaroside displays binding with *Ld*PPDK, which involves eight hydrogen bonds with Asn^366^, Gly^367^, Arg^369^(3), Arg^395^, Gln^400^ and Lys^897^ and other hydrophobic interactions are represented via Asp^346^, Lys^368^, Ala^374^, Glu^391^ and Leu^394^ (*n*=5) as represented in Ligplot (Supplementary Figure S5B). Cynaroside interacts with Gly^322^, Glu^44^ binding site residues of *Ld*TS by three intermolecular hydrogen bonds and hydrophobic interactions through residues Phe^19^, Lys^36^, Tyr^40^, Ser^42^, Thr^281^, His^284^, Met^288^, Glu^289^, Ala^292^, Trp^318^, Tyr^324^, Asp^605^ (*n*=12) as shown in Ligplot (Supplementary Figure S5C). The minimum binding energy of −10.4 kcal/mol and the value of predicted inhibitory constant of the Cynaroside with respect to *Ld*UGM was found to be 7.65 µM, which could be considered as its leishmanicidal activity (Table 1). Further, MD simulation of the highest affinity target, UDP galactopyranose mutase (UDPGM) upon cynaroside has been performed for 50 ns to understand the mechanism of interaction. After analyzing MD trajectories (using RMSD, RMSF, RG and SASA), we found that cynaroside perfectly bound into the active site UDPGM (Figure 6A–D). However, the trend of slight fluctuation continues in the cynaroside complexes till the end of simulation.

### Pharmacodynamic studies showed non-toxic properties of cynaroside

The pharmacological studies were done on cynaroside for a good oral administration established through the Lipinski’s rule of five, which was evaluated by Molsoft L.L.C.: Drug-likeness and molecular property prediction. Cynaroside followed all the parameters of Lipinski’s rule of five, except HBD and HBA criteria which are exceeding by 2 and 1 atoms, respectively. The lipophilicity of cynaroside showed value of 0.47 that indicates moderate sublingual absorption as observed from Table 2. Lipinski’s ‘rule of five’ [35] is an analytical approach for predicting drug-likeness stating that molecules had molecular weight (M.W. ≤ 500 Da), high lipophilicity expressed as LogP (LogP ≤ 5), hydrogen bond donors (HBD ≤ 5) and hydrogen bond acceptors (HBA ≤ 10) have good absorption or permeation across the cell membrane. For choosing the selected drug molecules through virtual screening, sometimes we found violation of some selection rules like Ro5, Veber etc. At present, in drug industry there are several important drugs available in the market which violate some likeness rules. Among the very popular drugs, some like fosinapril, bromocriptine mesylate, dabigitranetexilate, olmesartanmedoxomil and reserpine etc revealed two Ro5 rule violations [36]. The SwissADME was used for pharmacodynamic study of cynaroside to understand the action of drug inside a host’s body. Cynaroside possess low gastrointestinal absorption and good solubility with value −3.65, which is higher than −4 (≥ −4) [37]. Cynaroside is not permeable to the blood–brain barrier. The leadlikeness criteria is violated only for molecular mass (≥350) and possess a moderate bioavailability score. The results are summarized in Table 2. The ADMET study focused on the parameters that can define absorption, distribution, metabolism, excretion, toxicity, gastrointestinal absorption (GIA), solubility (LogS), P-glycoprotein substrate inhibition, cytochrome substrate/inhibitor. Cynaroside was evaluated as an active enzyme inhibitor with value 0.42. The predicted bioactivity by molinspiration is shown in Table 3. Molinspiration was used to evaluate the bioactivity of cynaroside by calculating the activity against GPCR ligand, kinase inhibitor, ion channel modulator, protease inhibitor, nuclear receptor ligand and enzyme inhibitor [37]. The bioactivity values were interpreted as follows: inactive (bioactivity score ≤ −5.0), moderately active (bioactivity score: −5.0 to 0.0) and active (bioactivity score ≥ 0) [38]. As per the Organisation for Economic Co-operation and Development (OECD) chemical classification, cynaroside was found to be a non-toxic as mentioned in Table 4. The principal aim of predicting the acute toxicity is to evaluate undesirable side effects of a compound after single or multiple exposures to an organism via a known administration route (oral, inhalation, subcutaneous (sc), intravenous (iv) or intraperitoneal (ip)). GUSAR was used to determine the acute toxicity of the successfully docked cynaroside based on the Prediction of Activity Spectra for Substances algorithm and Quantitative Neighborhoods of Atoms descriptors. The obtained result was compared with Toxicity Database to categorise on the basis of OECD chemical classification manual [28]. The criteria used for cynaroside to elicit toxicity based upon the administration route when the compound dose is more than 7000 mg/kg for intravenous route, more than 500000 mg/kg in case of the oral route, and more than 20000 mg/kg for intraperitoneal route and subcutaneous database as shown in Table 4. All the predicted toxicity risk factors for cynaroside were low and molecular weights less than 500, implied that it is likely to be absorbed and are capable to reach the place of action when administered as drugs [39]. It was predicted that cynaroside possessed no mutagenic, tumorigenic, irritant and reproductive effective toxicity risks as shown in Table 5.

**Table 2:** Evaluation of physico-chemical and ADMET properties of the ligands after docking

Ligand	MW (<500)	HBD (<5)	HBA (<10)	Number of rotatable bonds	miLogP	Drug likeness (Lipinski violations)	Lead likeness	Molar refractivity	TPSA (Å^2^)	Skin permeation (Log*K*_p_) (cm/s)	GI- absorption	BBB permeant	CYP1A2 inhibitor	Bioavailability score	Water solubility (Log S)
Cynaroside	448.4	7	11	4	0.19	Yes; 2 violations	No; 1 violation: MW > 350	108.13	190.28	−8.00	Low	No	No	0.17	−3.65 (Soluble)

**Table 3:** Bioactivity prediction of the selected inhibitor against *L. donovani* by molinspiration

Ligand	GPCR ligand	Ion channel modulator	Kinase inhibitor	Nuclear receptor ligand	Protease inhibitor	Enzyme inhibitor
Cynaroside	0.09	−0.02	0.15	0.27	−0.01	0.42

**Table 4:** *In silico* prediction of acute toxicity in rodent models and chemical classification of selected medicinal compounds

Ligand	Rat oral LD_50_ (mg/kg)	Rat iv LD_50_ (mg/kg)	Rat sc LD_50_ (mg/kg)	Rat ip LD_50_ (mg/kg)	OECD chemical classification
Cynaroside	2004000	3389000	5767000	691600	Non-toxic

Abbreviation: LD_50_, lethal dosage 50.

**Table 5:** Toxicity risks assessment of phytocompounds predicted by OSIRIS property explorer

Ligand	Mutagenic	Tumorigenic	Irritant	Reproductive effect
Cynaroside	No	No	No	No

## Conclusion

Here, it was observed that cynaroside has the potential antileishmanial activity. It showed better response when used in combination with low concentrations of miltefosine. Through *in silico* study, we have found that the binding energy and the binding site residues of the *Ld*UGM exhibited best interaction with the inhibitory flavonoid, cynaroside. It was confirmed by MD simulation study also. Our results suggested that cynaroside may be used as food constituent after the detailed *in vivo* studies on experimental visceral leishmanaisis, to fight against leishmaniasis.

## Supplementary Material

Supplementary Figures S1-S5 and Tables S1-S3

## Data Availability

Data are with the authors and will surely be provided on request through corresponding author.

## Abbreviations

ADMET: absorption, distribution, metabolism, excretion and toxicity
CADD: computer-aided drug designing
CC_50_: cytotoxic concentration 50%
DMSO: dimethyl sulfoxide
FBS: fetal bovine serum
HBA: hydrogen bond acceptor
HBD: hydrogen bond donor
H_2_DCFDA: 2,7-dichloro dihydro fluorescein diacetate
IC_50_: 50% of inhibitory concentration
ip: intraperitoneal
iv: intravenous
*K*_i_: lowest inhibition constant
MD: molecular dynamics
MTT: 3-(4,5 dimethyl- thiazol-2-yl)-2,5-diphenyl tetrazolium bromide
OECD: Organisation for Economic Co-operation and Development
PDB: Protein Data Bank
PPDK: pyruvate phosphate dikinase
PTR1: pteridine reductase1
RMSD: root-mean-square deviation
ROS: reactive oxygen species
sc: subcutaneous
UDP: uridine diphosphate
UDPGM/UGM: UDP galactopyranose mutase
