# Supplementary material for: Cynaroside inhibits Leishmania donovani UDP-galactopyranose mutase and induces reactive oxygen species to exert antileishmanial response
Source: Biosci Rep. 2021 Jan 12;41(1):BSR20203857. doi: 10.1042/BSR20203857 (PMC7805024; doi:10.1042/BSR20203857)
Supplement: Supplementary Figures S1-S5 and Tables S1-S3 [file BSR-2020-3857_supp.pdf]

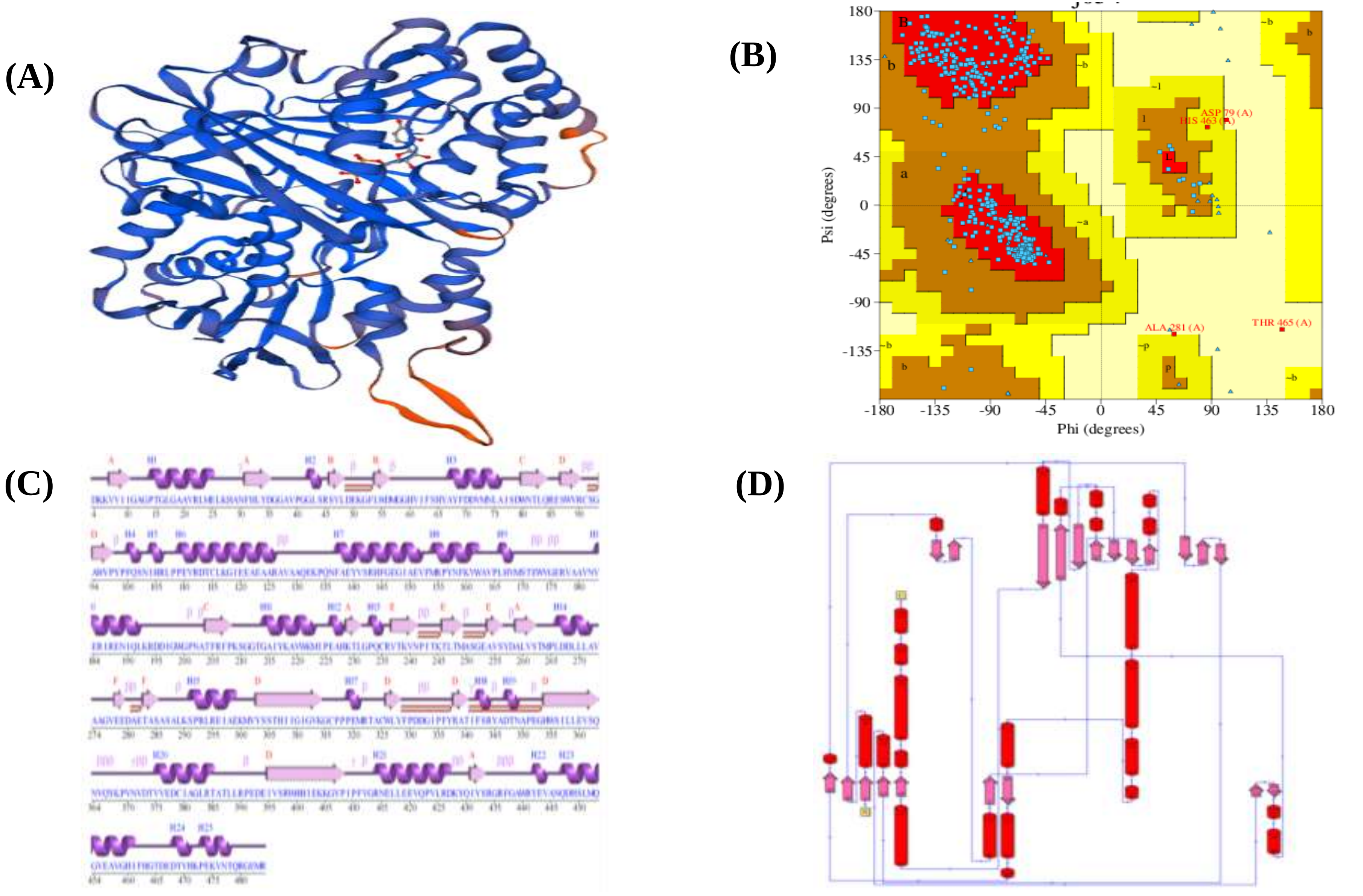
(A)
(B)
(C)
(D)
Psi (degrees)
Phi (degrees)
180
135
90
45
0
-45
-90
-135
-180
ASP 79 (A)
HIS 463 (A)
ALA 281 (A)
THR 465 (A)

Fig. 1.

S1 Fig: Homology modelling and structure validation of *Ld*UGM: (A) Cartoon representation of ***Ld*UGM** 3D structure **(B)** Ramachandran plot of UDP galactopyranose mutase showing 99.4% residues are in the allowed region. **(C)** Secondary structure elements and **(D)** topology map of UDP galactopyranose enzyme, indicating a conserved α and β framework.

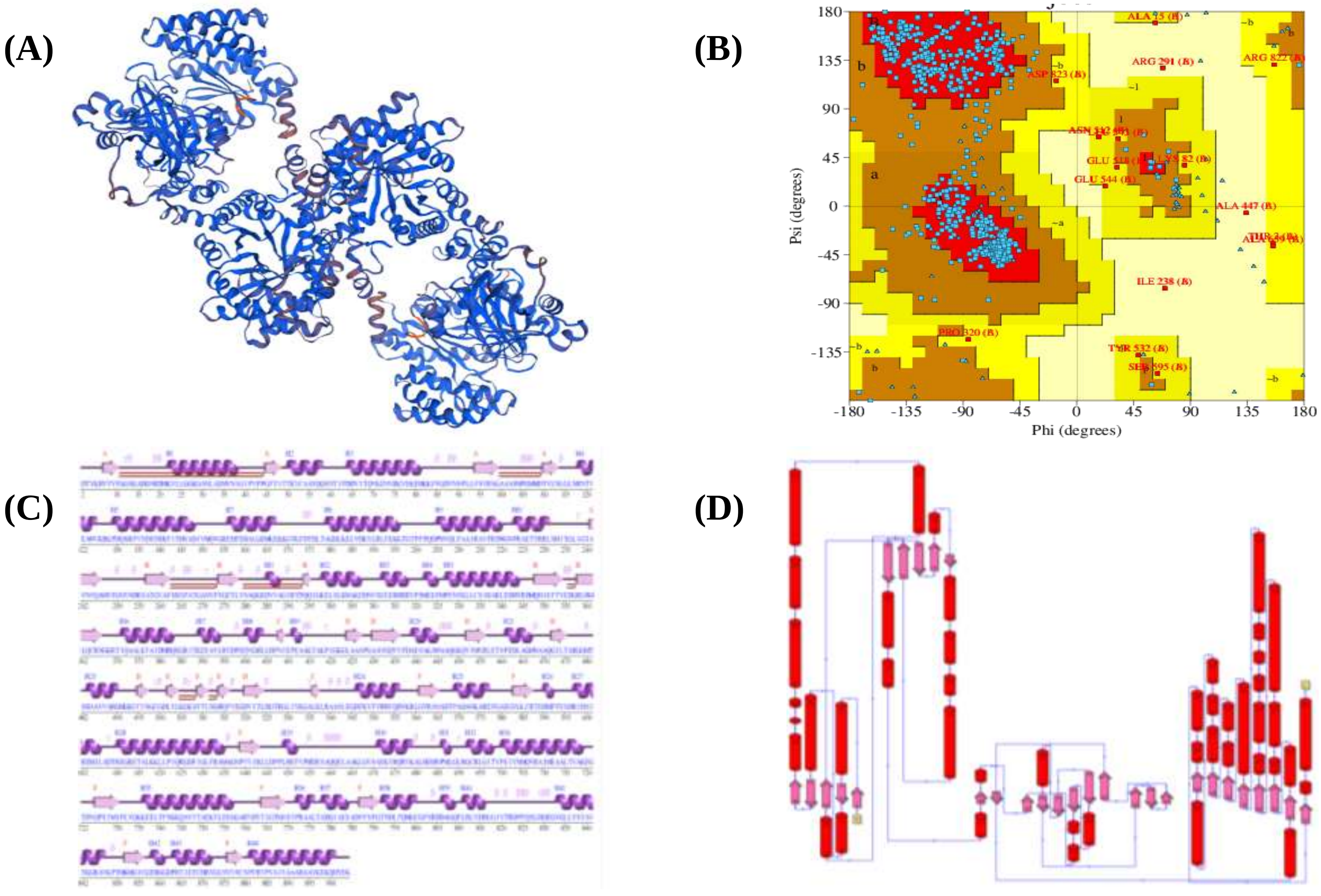

(A)
(B)
(C)
(D)
180
135
90
45
0
-45
-90
-135
Psi (degrees)
-180
-135
-90
-45
0
45
90
135
180
Phi (degrees)
ALA 75 (B)
ARG 291 (B)
ARG 822 (B)
ASP 823 (B)
ASN 532 (B)
GLU 544 (B)
ALA 447 (B)
ILE 238 (B)
PRO 320 (B)
TYR 532 (B)
SER 595 (B)

Fig. 2.

S2 Fig: Homology modelling and structure validation of *Ld*PPDK: (A) Cartoon representation of Pyruvate phosphate dikinase 3D structure **(B)** Ramachandran plot of Pyruvate phosphate dikinase showing 98.3% residues are in the allowed region **(C)** Secondary structure elements and **(D)** topology map of Pyruvate phosphate dikinase enzyme, indicating a conserved α and β framework.

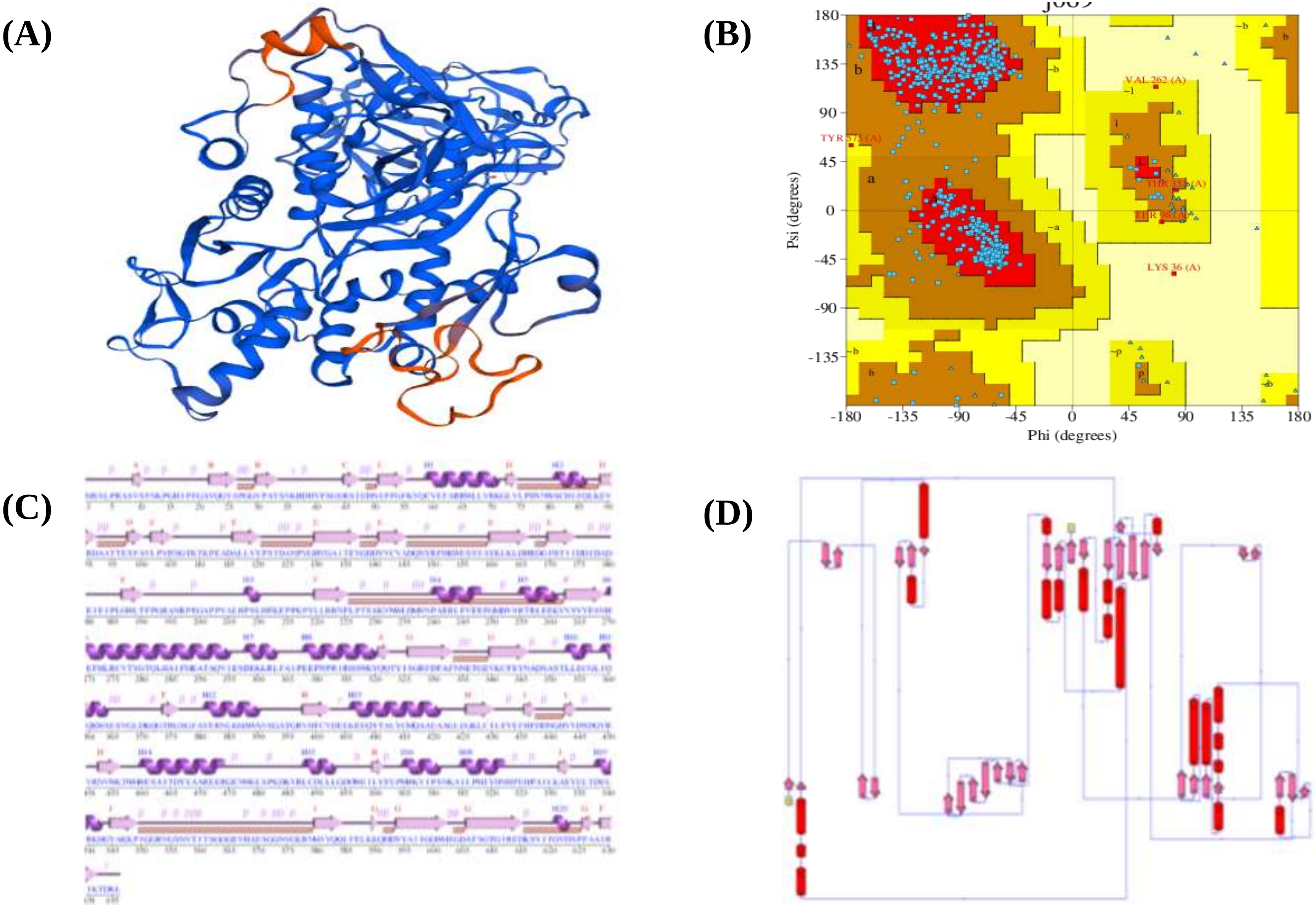
(A)
(B)
(C)
(D)
180
135
90
45
0
-45
-90
-135
-180
-135
-90
-45
0
45
90
135
180
Psi (degrees)
Phi (degrees)
VAL 262 (A)
TYR 575 (A)
LYS 36 (A)

Fig. 3.

S3 Fig: Homology modelling and structure validation of *Ld*TS (A) Cartoon representation of *Ld*TS 3D structure and **(B)** Ramachandran plot of *Ld*TS showing 99.4% residues are in the allowed region. **(C)** Secondary structure elements and **(D)** topology map of *Ld*TS enzyme, indicating a conserved α and β framework

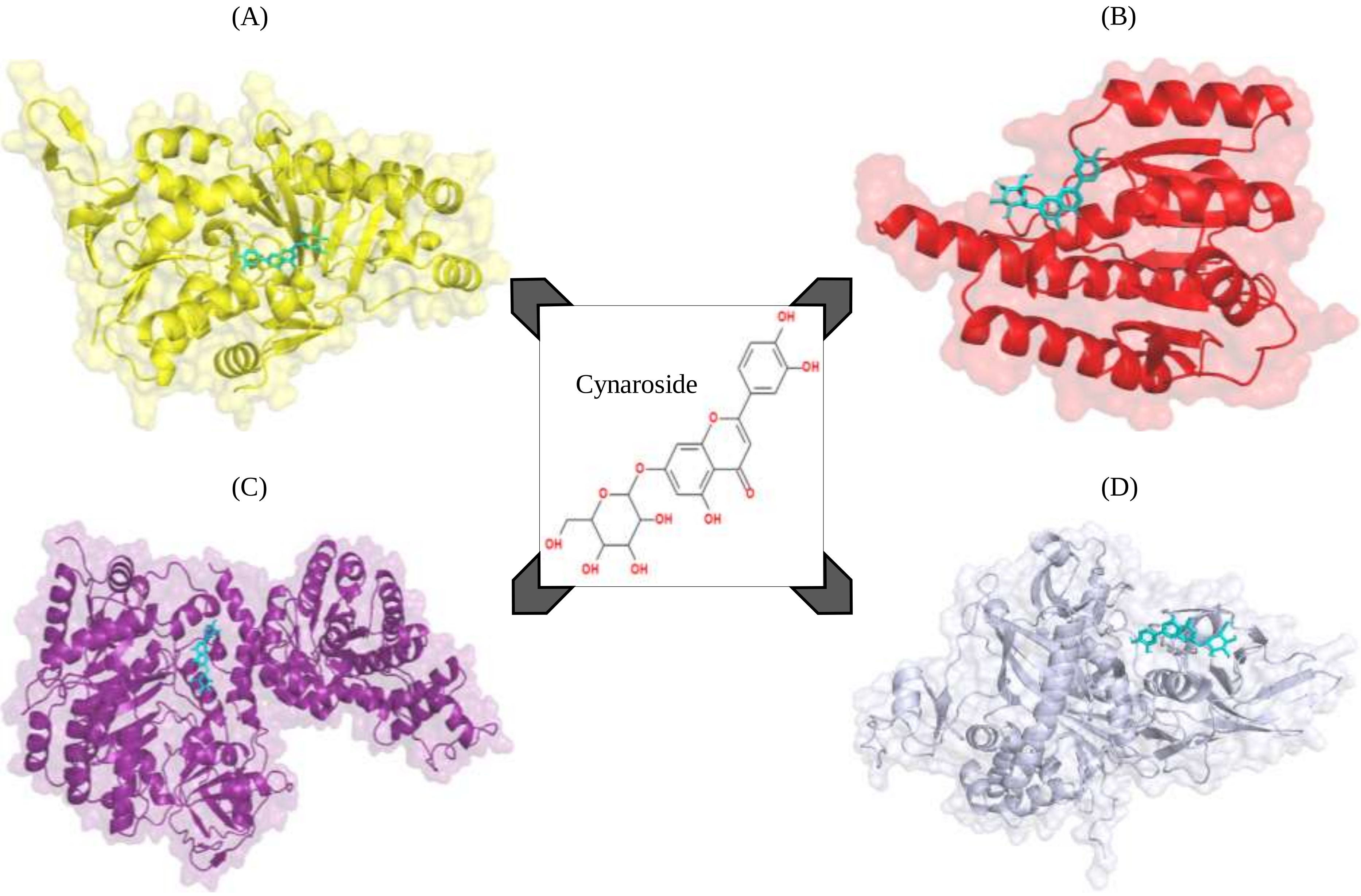

Fig. 4.

S4 Fig: Ligand docking with *L. donovani* important drug-targets: The cartoon-surface representation of target proteins with cynaroside (stick figure in cyan color) after docking as **(A)** *Ld*UDP **(B)** *Ld*PR1 **(C)** *Ld*PPDK and **(D)** *Ld*TS.

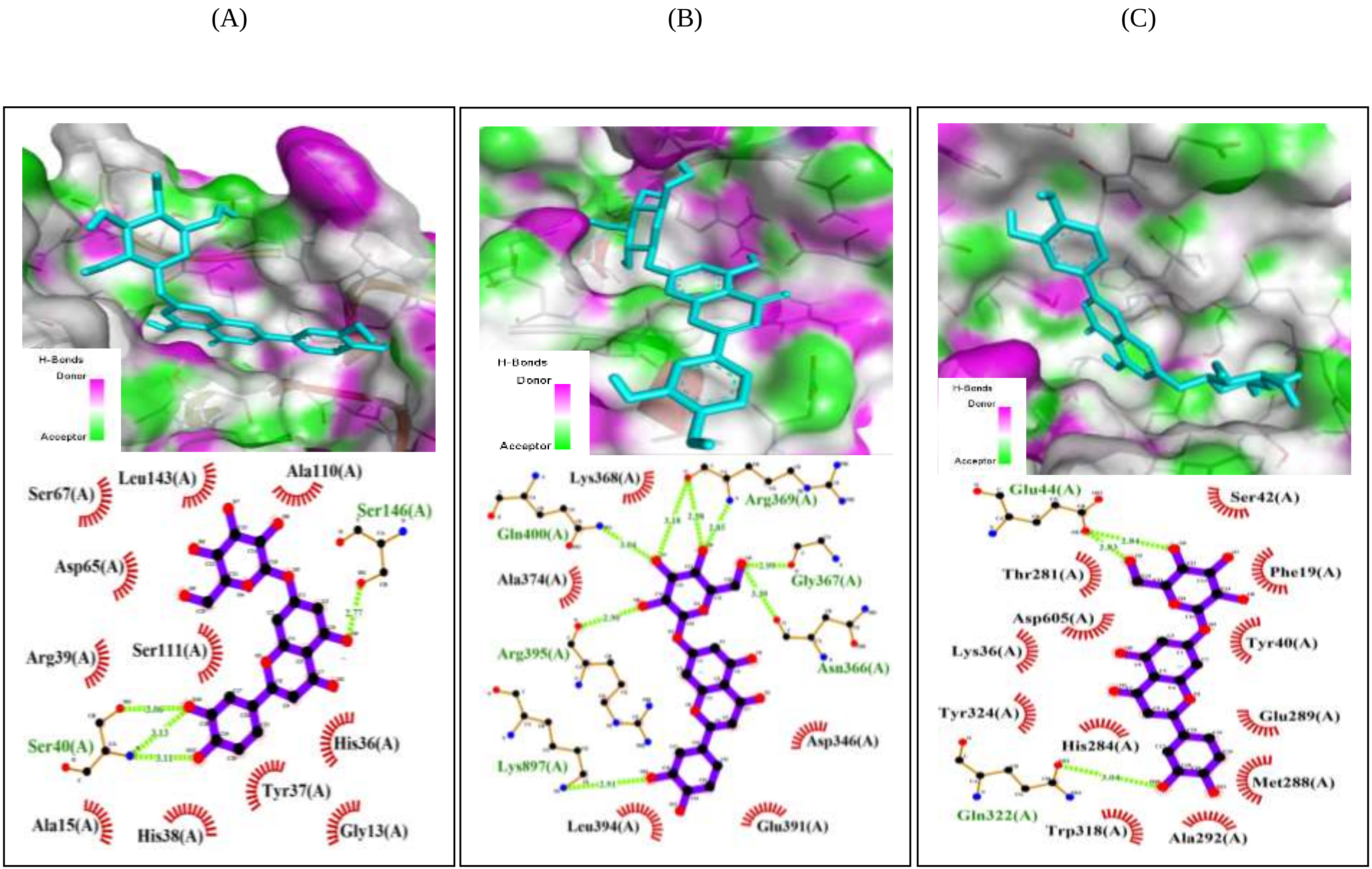
(A)
(B)
(C)
H-Bonds
Donor
Acceptor
Ser67(A)
Leu143(A)
Ala110(A)
Ser146(A)
Asp65(A)
Arg39(A)
Ser111(A)
Ser40(A)
His36(A)
Tyr37(A)
Ala15(A)
His38(A)
Gly13(A)
H-Bonds
Donor
Acceptor
Lys368(A)
Arg369(A)
Gln400(A)
Ala374(A)
Gly367(A)
Arg395(A)
Asn366(A)
Lys897(A)
Asp346(A)
Leu394(A)
Glu391(A)
H-Bonds
Donor
Acceptor
Glu44(A)
Ser42(A)
Thr281(A)
Phe19(A)
Asp605(A)
Lys36(A)
Tyr40(A)
Tyr324(A)
Glu289(A)
His284(A)
Met288(A)
Gln322(A)
Trp318(A)
Ala292(A)

Fig. 5.

S5 Fig: Molecular view of cynaroside *L. donovani* selected drug-targets: (A) *Ld*PR1 **(B)** *Ld*PPDK and **(C)** *Ld*TS. Amino acid residues forming hydrophobic interactions were highlighted in red circles. Amino acids contributing to hydrogen bonds were labelled dark green and hydrogen bonds were indicated as dotted lines with bond length labelled in lime green.

S1 Table: PDB ID and template structures used for homology modelling of target proteins of *L. donovani.*

Sl. No.	Enzymes Name	Template PDB ID used for modelling	Template structure
1.	UDP galactopyranose mutase	4DSH	Crystal structure of reduced UDP-Galactopyranose mutase from *Trypanosoma cruzi*
2.	Pyruvate phosphate dikinase	2X0S	3.0 a resolution crystal structure of glycosomal pyruvate phosphate dikinase from *Trypanosoma brucei*
3.	Trypanothione synthetase	2VPM	Trypanothione synthetase from *Leishmania major*

S2 Table: List of parameters used for the validation of homology modelled key drug-targets of *L. donovani*.

**Enzymes**	**Sequence identity**	**GMQE**	**Q-Mean**	**% of residue in Ramachan--dran favoured region**	**% of residue in Ramachan--dran allowed region**	**% of residue in Ramachan--dran outlier region**	**RMSD value**
UDP Galacto pyranose mutase	59.87%	0.76	-1.97	96.9%	2.5%	0.6%	0.237
Pyruvate phosphate dikinase	75.93%	0.85	-1.25	93%	5.3%	1.7%	0.329
Trypano-thione synthetase	96.47%	0.95	-0.96	95.7%	3.6%	0.6%	0.118
